# Silicone Oil: Different Physical Proprieties and Clinical Applications

**DOI:** 10.1155/2014/502143

**Published:** 2014-06-11

**Authors:** Francesco Barca, Tomaso Caporossi, Stanislao Rizzo

**Affiliations:** U.O. Chirurgia Oftalmica, Azienda Ospedaliero Universitaria Pisana (AOUP), Via Paradisa 2, 56124 Pisa, Italy

## Abstract

Silicone oils are important tools in vitreoretinal surgery because they have the ability to displace aqueous humor from the retinal surface, maintaining the adhesion between retina and retinal pigment epithelium. To understand this capability, it is important to know the silicone oil characteristics. Herein, we report first on the main chemical-physical proprieties and then we review the clinical applications of the current silicone oil which is lighter than water with particular reference to their indications with small gauge vitrectomy. Finally, we describe the surgical techniques to inject and remove this type of silicone oil. In the summary of this paper, we explain why silicone oils are today increasingly used and why their introduction has improved the prognosis of several retinal diseases. In fact, having different types of silicone oils allows us to choose the appropriate endotamponade for every single case.

## 1. Introduction

Silicone oils are important tools in vitreoretinal surgery and their introduction has represented a pivotal moment in the management of ophthalmic surgery as they are equipped with a combination of chemical and physical properties that have propelled their surgical use [1]. Silicone oils are essentially used as intraocular tamponade thanks to their ability to maintain the adhesion between retina and retinal pigment epithelium (RPE).

The safe and effective use of tamponade substances means the knowledge of their physical and chemical properties because it is on the basis of this knowledge that surgeons have to decide what type of tamponade they should use.

## 2. Physical Properties of Tamponades and Clinical Consequences

To be effective as an internal tamponade, a silicone oil has to have the ability to displace aqueous humor from the retinal surface. The following 4 physical parameters influence this function [2].

*(1) Specific Gravity (SG)*. This explains why an intraocular tamponade sinks or floats in aqueous humor. Any substances with an SG of 1 are neutrally buoyant in water, those with SG greater than 1 are denser than water and so will sink in it, and those with an SG of less than 1 are less dense than water and so will float. The specific gravity of aqueous humor and vitreous humor is a little higher than that of water (SD 1.00). Since the specific gravity of silicone oils in comparison is a little lower (0.97), they float in vitreous cavity.

*(2) Buoyancy*. An intraocular bubble of tamponade agent is acted upon by two opposing forces: buoyancy (upward force) and the gravity on the bubble (downward force). Archimedes' principle indicates that the upward buoyant force that is exerted on a body immersed in a fluid, whether fully or partially submerged, is equal to the weight of the fluid that the body displaces. Archimedes' principle is a fundamental physics law of fluid mechanics. Regarding the vitreous cavity, the result is the force with which the bubble presses against the retina. For silicone oil, the “pressing” force is relatively small, as the specific gravity is close to that of aqueous humor. The force is greatest with air or gas, as the specific gravity is very low at 0.001.

*(3) Interfacial Tension.* When two immiscible agents are used together (e.g., silicone oil and aqueous humor), the interaction that occurs at the surface of these substances involved is named interfacial tension. Interfacial tension is a physical rating of the difference between the intermolecular force of the two liquids and it is responsible for the shape of liquid bubbles. Therefore, a substance with a high interfacial tension will have a greater tendency to stay as one large bubble without dispersion into small bubbles. Gas or air has the highest interfacial tension against water (around 80 mN/m), whereas perfluorocarbon liquids (PFCLs) and silicone oils have a lower interfacial tension, around 40–45 mN/m and 35 mN/m, respectively.

*(4) Viscosity*. The viscosity is the physical property of a fluid which measures its resistance to gradual deformation by shear stress. The tendency of a substance to emulsify and disperse into droplets over time is also dependent on its viscosity. The less viscous a substance, the lower the energy that is required to disperse a large bubble of the substance into small droplets. Silicone oils have a high viscosity (1.000–5.000 cs) and, once dispersed, the small droplets will tend to recoalesce back as a large bubble.

## 3. Chemical Properties

Silicone oil is a term generally used to describe a group of hydrophobic polymeric and monomeric compounds constituted of silicon-oxygen bonds and named organosiloxane [3]. Because of their viscosity and their ability to repel water, they are referred to as oils.

Silicone oils are constituted of a linear chain of siloxane repeating units (–Si–O) and a variety of side chains (radical side groups). Those used in ophthalmology have hydrocarbon radicals as radical side groups (e.g., methyl, phenyl, vinyl, and trifluoropropyl groups). These composts are attached to the silicon atom and it is possible to have many different combinations. Therefore, one silicon atom can bond two radical groups of the same type (e.g., dimethyl-siloxane) or two different groups (e.g., phenyl-methyl-siloxane).

The major differences among silicone oils depend on the molecular weight (MW), on the length of the linear chain, and on the chemical structure of radical side groups, radical end termination of the polymer chains, and the size distribution of the chain. Thus, each type of silicone oil has specific chemical and physical characteristics.

The viscosity of different types of silicone oil, which is expressed in centistokes (1 cs = 10^−6 ^m^2^/s), arises from the molecular weight and from the length of the polymers: increasing a silicone oil's molecular weight results in an increased polymer chain length and consequently an increase in its viscosity. Silicone oils currently used have a viscosity ranging from 1.000 (MW 37 kDa) to 5.000 cs (MW 65 kDa).

In order to create a tamponade effect on the superior or inferior retina, silicone oils have the capability to be lighter or heavier than water and this property arises from the radical side groups. Herein, we review the major indications regarding the use of the first generation of silicone oils, which is named lighter than water. They are the most commonly used and are referred to as polydimethylsiloxane (PDMS). Their viscosity rating is between 1000 and 30.000 cs.

## 4. Physical Properties

The aim of the use of silicone oil as vitreous substitute is to provide short- to long-term tamponade of the retina. The dynamic of the silicone oil depends on the interaction between buoyancy, interfacial surface tension, and viscosity.

Buoyancy arises from the difference in specific gravity between aqueous (or vitreous) humor and the selected silicone oil. As we have seen at the beginning of this paper, the specific gravity determines whether a vitreous substitute will sink or float in aqueous humor. If compared with water (specific gravity of 1.00), the specific gravity of aqueous humor and vitreous humor is a little higher than this while the gravity of silicone oil is a little lower (0.97). Therefore, silicone oil floats inside the vitreous cavity and the upward force is defined as buoyancy. This force is highest at the apex and gradually decreases to zero at the horizontal meniscus. Consequently, tamponade force arises from the difference in density between aqueous humor, vitreous humor, and silicone oil bubble. However, the buoyancy does not act upon a single point but is spread over a limited area and, for this reason, it produces pressure (force/unit area) [3].

Surface tension is responsible for the shape of liquid droplets because it describes the forces that tend to keep a bubble whole. In general, for 1000 cs silicone oil, it is 40 mN/m (at 25°C) that is approximately one-third of that generated on an air bubble. There are several factors that may influence the surface tension of a silicone oil bubble once it is injected into the eye. First, the viscosity: the higher the viscosity, the higher the surface tension. This is one of the reasons why silicone oils with higher viscosity are considered to emulsify less frequently than silicone oils with lower viscosity. There are then many factors that may decrease the surface tension: viscoelastic solutions, blood, proteins, lipids, and ionized solutions (e.g., biological fluids) are factors that, if present in the vitreous cavity when a silicone oil is injected, can decrease the surface tension and therefore lead to emulsification.

## 5. Does Viscosity Make a Real Difference in Emulsification Rate?

A major permanent problem in the use of silicone oils as vitreous substitutes is their tendency to emulsify. Emulsification means the formation of small oil droplets at the interface between oil bubble and intraocular fluids or tissues and it causes a dispersion of these droplets into the aqueous humor and vitreous humor with consequently higher risk of proliferative vitreoretinopathy, failed retinal detachment, inflammation, secondary glaucoma, and keratopathy, even after silicone oil removal [4, 5].

In fact, once coated by emulsifiers, the droplets remain dispersed, pass through retinal breaks or through the zonules into the anterior segment, and cause inflammation and activation of neutrophils [6].

The tendency to emulsify depends on several factors: interfacial surface tension, viscosity, chemical composition, content of low molecular weight (MW) siloxane compounds or other impurities, and absorption of various biological substances from intraocular fluids and tissues (named emulsifiers) have been studied and all of them can have a role in the emulsification process [3].

The presence of a low MW siloxane compounds is very critical. For a given viscosity, the silicone oil with the lowest MW average will emulsify faster, while a purified silicone oil with higher average MW will exhibit a better biocompatibility and a higher resistance to the emulsification and therefore the removal of low MW compounds during the purification process of a silicone oil is very important.

Since a silicone oil with a higher viscosity has a lower tendency to emulsify, many surgeons prefer these types of silicone oil, such as 5000 cs, especially when the oil is intended to serve as a prolonged or permanent tamponade. However, although there are several studies demonstrating in vitro that the increasing viscosity of silicone oil reduces the tendency to emulsify [7–9], the commercially available 1000 cs and 5000 cs silicone oils do not have a clinically significant difference in emulsification [10]. Despite their difference in viscosity, the 1000 cs and 5000 cs silicone oils have nearly the same behavior because in reality there are many other determinant factors influencing the tendency to emulsification: first, because, despite marked viscosity difference, the 1000 cs and the 5000 cs silicone oils have nearly the same surface tension (21.2 mN/m and 21.3 mN/m, resp.) [3]; second, because, although the commercially available preparations of 1000 cs and 5000 cs silicone oils have reached a high grade of purity to minimize the risk of silicone oil emulsification, the presence of impurities in the oil, such as low MW molecules, may still occur; third, because, once inside the vitreous cavity, silicone oil adsorbs biological solutes from ocular fluids, blood, or tissue such as lipoproteins, cholesterol, and retinol [2, 3]. Each of these components is an emulsifier because the contact of these substances with the silicone oil bubble decreases their surface tension and consequently increases the tendency to emulsify. Therefore, it is easy to understand why in cases of hemorrhages and inflammation or rather when the concentration of these emulsifiers is high the risk of silicone oil emulsification is higher.

Emulsification has been reported to occur after several months. In a study of 150 eyes, Federman and Schubert [5] found that emulsification of silicone oil occurred in 1% after 1 month, 11% at 3 months, 85% at 6 months, and 100% after 12 months. The emulsification seems to be therefore time dependent [11] and it has been speculated that it depends on a combination of the saccadic motion, the difference in density of intraocular fluids and silicone oil, and the gradual decrease of interfacial surface tension of the oil due to the adsorption of surface-active components from the intraocular fluids [3, 8, 9].

With the advent of the Micro-Incisional Vitreoretinal Surgery (MIVS) surgeons prefer less viscous silicone oils which can be introduced and removed more easily through the small instruments and cannulae. However, a more viscous silicone oil would be less likely to emulsify. For this reason, new silicone oils with an increasing extensional viscosity have been studied [12–15]. These types of silicone oils are obtained by the addition of a small amount (around 5–10%) of very-long-chain silicone molecules to a common silicone oil. The advantage of a silicone oil with an increasing extensional viscosity would seem to be an increased resistance to emulsification while maintaining a low viscosity and therefore an easier injection and removal with small gauge instrumentation with respect to single grade oils of the equivalent shear viscosity. To date, there are only few publications about the use of these new types of silicone oils and all of them are in vitro tests. The main concept raised from those tests is that silicone oil blends containing small percentages of a high molecular weight of the same chemical composition as the bulk oil are more resistant to emulsification and are easier to inject than single grade oils of the equivalent shear viscosity. However, there is only a case report on their clinical use by Maier et al. in which they reported two cases of early emulsification with a 2000 cs silicone oil (Siluron 2000, Fluoron, Neu-Ulm, Germany) [16]. Therefore, there is no evidence yet in the literature whether these new types of silicone oil show lower tendency to emulsify and therefore further clinical studies are needed.

## 6. Potential Complication Related to the Postoperative IOP Control

Since a variety of complications are possible in case of a vitreoretinal surgery, it is important to understand whether they are associated with the use of silicone oil or related to the underlying pathology and other aspects of surgical intervention.

One of the main issues regarding the use of silicone oils is the management of the postoperative IOP. In fact, a postoperative IOP rise is not uncommon after vitrectomy with silicone oil injection. In the Silicone Study a chronic elevated IOP has been reported in the 8% of eyes treated with conventional silicone oil at 36 months [17]. However, in the literature, the incidence ranges from 2 to 40% [10, 18]. The causes of raised IOP are multifactorial but, schematically, there are 3 types of mechanisms.

1. Pupillary block glaucoma: it may develop at any time but it is more frequent in the early postoperative time (days to some weeks). Aphakic eyes have more risk compared to phakic/pseudophakic eyes. In either case, the block originates when aqueous humor cannot move in the anterior chamber due the presence of silicone oil bubble with consequent aqueous misdirection, shallow anterior chamber, and raised IOP. Therapy is to perform an inferior iridectomy (or reopen it) with YAG laser or tPA injection. Otherwise, a second operation may be considered.
2. Overfill of silicone oil: also this condition is more frequent in the early postoperative period and it is usually well treated with medical management. However, if the IOP remains too high, another operation with partial removal of silicone may be necessary.
3. Chronic elevation of IOP: while the first two mechanisms were related to the presence of a whole silicone oil bubble, a chronic elevation of the IOP is usually related to a silicone emulsification and consequent migration of silicone oil drops into the anterior chamber angle. The emulsified droplets may obstruct the trabecular meshwork and develop a trabeculitis. For this reason, the therapy of this complication consists in topical or periocular steroids, conventional antiglaucoma drops, and, eventually, the removal of silicone oil with careful attention to remove emulsified droplets from the anterior chamber. Unfortunately, when the trabecular meshwork is permanently damaged, the IOP does not return into a normal range and therefore a glaucoma surgery may be required. In this regard, glaucoma drainage devices have been investigated for the treatment of refractory glaucoma. Ishida et al. found that the chronically raised IOP can be well controlled using the Ahmed glaucoma valve even if the presence of silicone oil was associated with an increased risk of surgical failure when compared with eyes that had not been treated with silicone oil [19]. However, in a case-control study by Wong et al., the authors found that the use of a heavy silicone oil (Densiron-68) was associated with a higher IOP in the early postoperative period when compared with a conventional silicone oil [20].

## 7. Indications for Silicone Oil Tamponade

The history of silicone oil in ophthalmic surgery is very short in comparison with gases. In the USA, in fact, silicone oil as intraocular tamponade has been approved by the Federal Drug Administration only in 1996. Since that date its use has increased very fast. The first indications were complicated retinal detachment due to PVR or viral retinitis, giant retinal tears, trauma, and severe proliferative diabetic retinopathy [21, 22]. New possible indications are now retinal detachment due to macular hole in highly myopic eye [23, 24], chronic and persistent macular hole, colobomatous retinal detachment [25], and chronic uveitis with hypotony [26]. In summary, every time a long-term tamponade is required. Silicone oil has in fact the important advantage of determining a long support until the recovery of the retina has occurred. In case of retinal detachment, we usually perform a silicone oil removal after 3–6 months because we believe this time is sufficient for the recovery of the eye with minimal risk for the development of a PVR.

Silicone oil is also the first choice for patients that have to fly or for patients that cannot maintain the correct postoperative positioning such as children or old patients.

The Silicone Oil Study, a prospective, multicentered, randomized, controlled clinical trial comparing silicone oil and long-acting gases in the management of eyes with severe PVR, concluded that silicone oils were superior to sulfur hexafluoride gas (SF6) and equivalent to perfluoropropane gas (C3F8). If we consider however certain subgroups, it appears clear that the use of silicone oil was better in case of relaxing retinotomy, severe anterior PVR, difficulty in maintaining the postoperative positioning, and the need to fly or travel to higher altitudes. On the contrary, the use of C3F8 had the relative indications for those cases with a poor iris diaphragm due to a high probability of corneal tamponade touch; superior retinal breaks on the posterior edge of a scleral buckle, because gas conforms better than silicone oil to the slope of the buckle; and the presence of a silicone intraocular lens with an open posterior capsule [27–29].

The rationale for silicone oil use in the management of PDR is the reduction in postoperative hemorrhages and the presence of a severe anterior segment neovascularization [30]. This is because silicone oil may prevent the flow of vasoproliferative factors into the anterior segment decreasing the risk of iris rubeosis and neovascular glaucoma. Obviously, we also use silicone oil after failure of conventional vitrectomy for PDR due to the development of PVR.

The use of silicone oil for macular hole repair is very controversial. We never use silicone oil as the first tamponade of an idiopathic macular hole, except in some particular cases. We prefer gas for many reasons. Gas has a higher superficial tension and better buoyancy and does not need a second surgery to remove it. This technique is well established nowadays and the success rate with the use of gas is very high [31]. We consider the use of silicone oil for selected and particular cases in the treatment of idiopathic macular holes when there are problems with the postoperative positioning, air travel is necessary, or one-eyed patient.

Before the advent of PFCLs, silicone oil has been used in the treatment of giant retinal tears both as intraocular tool and as postoperative tamponade. As an intraocular tool, silicone oil has been used to facilitate unfolding and flattening of retinal tears and retinal detachment. Today, we prefer for this purpose using PFCLs because they are easier to use and because they unfold and flatten the retina less traumatically. As permanent tamponade, silicone oils are indeed very useful. While gases may be used to repair a superior giant tear, in all other cases, we prefer silicone oil as intraocular tamponade until we believe the retina recovered.

The advent and the improvement of the vitreoretinal surgery have improved the prognosis of traumatized eye [32]. Silicone oils are used to minimize the risk of postoperative bleeding, maintain retina attached, and avoid phthisis in case of severely traumatized eyes. Often, several operations are needed to obtain a reasonable anatomical and functional outcome. After silicone oil removal, the high failure rate is generally due to the development of a retinal detachment and/or PVR that requires a new operation. In this regard, since it is believed that the proliferative process continues for a longer period of time than in eyes with nontraumatic retinal detachment, especially in case of penetrating ocular trauma, it is recommended to leave silicone oil for several months.

## 8. Surgical Techniques

### 8.1. Silicone Oil Injection

Today, silicone oils are mainly used as intraocular tamponade because the recent introduction of PFCLs has decreased the use of silicone oils as intraoperative tool.

With the modern vitrectomy systems, the injection and the removal of silicone oils are performed using a syringe connected to a pump that is controlled by the surgeon with the foot-pedal. Since, according to Poiseuille's law, the flow of a fluid in a tube is proportional to the fourth power of the radius of the tube and inversely proportional to the length of the tube and due to the high viscosity and the high pressure that is required to infuse silicone oils into the vitreous cavity, special devices have been developed. The system is therefore made of a large syringe, an infusion line as short as possible, and nondistensible material. A large syringe is required because it has to handle the high infusion pressure, while a short infusion line is important to reduce the resistance during the injection and the removal. The use of a nondistensible material is important to avoid that, once the injection is stopped, undesired further injection occurs when the distended tube returns to its normal diameter, forcing more silicone into the eye [33].

There are 3 surgical techniques to inject silicone oils into the eye:

- fluid-silicone exchange,
- air-silicone exchange,
- perfluorocarbon liquid-silicone exchange (the so-called direct exchange).

We do not perform a fluid-silicone exchange because, due to the low surface tension, we believe that the risk is too high that silicone oil enters the subretinal space through retinal breaks. The choice of air, rather than perfluorocarbon liquid-silicone exchange, depends on two main reasons: the eventual presence of a retinotomy or an anterior break. If the break is in the mid periphery or at the posterior pole, we usually perform first a fluid-air exchange with internal drainage of subretinal fluid and then, once the retina is flattened under air, an air-silicone exchange. If a relaxing retinotomy has been applied or the retinal break is anterior, we prefer to perform a direct exchange between PFCL and silicone oil in order to avoid the slippage of the posterior edge of the tear. In this case, we connect the syringe to the infusion line and an extrusion needle is placed into the vitreous cavity. In this way, while the surgeon injects the silicone oil pushing the foot-pedal, either passive or active aspiration may be used to remove the PFCL. In case of 23- or 25-gauge vitrectomy systems, we set the machine with an aspiration rate from 0 to 30 mmHg and an infusion pressure rate from 0 to 28 Psi. It is important to not exceed 30 Psi to avoid the disconnection of the infusion line from the eye.

Regarding the management of the anterior segment, particular attention has to be drawn to aphakic patients. In these patients, an inferior peripheral iridectomy (IPI) is mandatory to avoid pupillary block secondary to the silicone oil filling. The IPI allows aqueous humor to pass under the silicone oil bubble and to enter into the anterior chamber without causing a pupillary block.

### 8.2. Silicone Oil Removal

Also, regarding silicone oil removal, there are several techniques. Some surgeons prefer to remove silicone oils using a two-port system, one for the infusion line and another one to aspirate the tamponade. Using this technique, it is also possible to passively remove silicone oil through a small cornea incision in aphakic eyes. At the end of the procedure, surgeons have to control whether retina is still attached using a binocular indirect ophthalmoscope. We do not use this technique anymore for several reasons: first, because it is impossible to have a direct control of the intraocular pressure and because it is impossible to be sure that the silicone oil has been removed completely. Finally, with this technique, it is impossible to remove the silicone oils which are heavier than water.

We therefore always perform a standard three-port pars plana vitrectomy also for silicone oil removal. We set the machine with an IOP at 20–25 mmHg and a vacuum rate from 0 to 650 mmHg. We currently use the new high-flow extraction sleeve by Alcon that significantly improves oil extraction compared with the old system, up to 5 times faster depending on the gauge. The sleeve consists of a silicone tube that is inserted on the head of the cannula (both 23- and 25-gauge) and allows the aspiration of the silicone oil through the cannula. To remove the sleeve from the cannula, it is enough to exert a tilt motion, taking care to keep the head of the cannula with a forceps (e.g., Bonn).

Once the silicone oil bubble has been removed, we perform several fluid-air exchanges in order to remove every small drop of silicone oil that remained inside the eye. In case of emulsified drops in the anterior chamber, their removal through a small corneal incision to minimize postoperative risk of complications such as silicone keratopathy and secondary glaucoma is mandatory.

In phakic eyes, the decision whether or not to remove the lens depends on the presence of a cataract and the patient's age. If there is a significant cataract, we usually remove the lens during vitreoretinal surgery. If the crystalline lens is clear, the decision arises from the age of the patient because in patients older than 50 years we prefer to remove the lens because the eye will develop a significant cataract few months after the surgery. In this case, we perform a combined phacoemulsification, posterior-chamber IOL implantation, and silicone oil removal.

## 9. Conclusions

Silicone oils are very useful surgical tools because they are able to simplify the surgical management of many vitreoretinal diseases. With the modern vitrectomy systems and the possibility to use such different tamponades, the prognosis of several diseases has improved. According to the vitreoretinal pathology, we can choose between a variety of silicone tamponades and we can therefore select the best intraocular tamponade in relation to the underlying disease.

Depending on the situation and the duration of the tamponade, we can decide which of the various characteristics of an endotamponade would be the most important. Nevertheless, we have to keep in mind that there are some steps, such as complete removal of any traction, that are crucial for the success of the surgery.

The decision of silicone oil usage may be taken both before and during the operation. The first and more important parameter of choice is the time that is, in surgeon's opinion, requested for the tamponade. Before the operation, it is fundamental to take into consideration the type of vitreoretinal disease (e.g., is there a PVR? And is the risk of a surgical failure high?) and the postoperative position because if we know that it is impossible for the patient (e.g., children) to maintain a certain postoperative positioning, maybe it is better to choose silicone oil instead of another tamponade. However, even if we have not programmed the use of a permanent tamponade, it is possible to decide during the operation that silicone oil is needed. For example, the decision to perform a retinotomy or complications such as an intraocular bleeding can indicate the choice of silicone oil.
